# Magnum metal-on-metal uncemented total hip replacement: 8- to 18-year outcomes of 211 cases

**DOI:** 10.1007/s12306-024-00831-3

**Published:** 2024-06-04

**Authors:** D. Gaillard-Campbell, T. Gross

**Affiliations:** Midlands Orthopaedics and Neurosurgery, PA, 1910 Blanding Street, Columbia, SC 29201 USA

**Keywords:** Total hip arthroplasty, Large bearing, Metal-on-metal, Trunnion corrosion

## Abstract

**Background:**

Reports of adverse reactions to metal debris contributed in part to a decline in use of large-bearing metal-on-metal total hip devices. We hypothesize an optimal trunnion design may reduce risk of this failure mode in large-bearing total hip arthroplasty systems. The purpose of this study is to report mid- to long-term outcomes for a single-surgeon series of 211 total hip arthroplasties using the large-bearing Biomet Magnum metal-on-metal system.

**Materials and methods:**

Between December 2004 and January 2016, the primary surgeon performed 211 uncemented Magnum total hip arthroplasties in 181 patients. The average length of follow-up was 10.1 ± 3.5 years (range 8–18 years).

**Results:**

Using failure of any component as the endpoint, the overall survivorship rate was 98.1% at 10 years and 97.4% at 18 years. These eight failures (3.8% of cohort) included one case of adverse wear-related failure (0.5%), two cases of acetabular ingrowth failure (0.9%), three cases of trunnion corrosion (1.4%), one failure of late infection (0.5%), and one inappropriate revision of components for trochanteric nonunion without instability (0.5%). Excluding failed cases, all components were radiographically stable with no radiolucencies. Except for the one wear failure, ion testing revealed that 97.2% of cases were within optimal whole blood metal ion levels with the remaining ion test results within acceptable levels.

**Conclusions:**

With the uncemented Magnum metal-on-metal total hip, we achieved 97.4% 18-year implant survivorship, exceeding the NICE criteria and registry benchmarks for implant survivorship. We observed a trunnion corrosion rate of 1.4% and no cases of instability. The single case of adverse wear-related failure was caused by acetabular component malposition.

## Introduction

Total hip replacement (THR) has continually grown in popularity and effectiveness since its modern conception in the early 1960s [1]. Despite its widespread use and rapid growth, there still exists a 5% failure rate at 10 years postoperatively in older patient populations; this registry-reported failure rate rises to 20% in younger patient populations [2–4]. Common issues still include a 2% rate of deep infection [5], a 3–5% rate of instability [6, 7], a 20% rate of residual unexplained pain [8, 9], suboptimal function in those desiring impact sports [10], and a currently unknown rate of trunnion corrosion [11, 12]. These issues do not negate the value and many benefits of THR, but they are worth exploring so that solutions might be developed. After the initial success of the Metasul small-bearing metal-on-metal (MoM) THR [13–15] and MoM hip resurfacing arthroplasty (HRA) [16–19], large-bearing THR became available from many manufacturers. These implants utilized a resurfacing-style acetabular component and added a matching femoral head that allowed connecting to a standard THR trunnion. The popularity of these bearings was fueled by low in vitro wear [20] and a lower risk of instability [21]. However, reports of failures due to adverse reaction to metal debris (ARMD) [22–24] began appearing with growing frequency, eventually leading to near-total abandonment of these devices.

Large-bearing MoM devices more closely mimic the natural hip with minimal instability [25], but these devices suffered from a high rate of ARMD [22, 24]. Unlike most THR cups (180° arc of coverage), resurfacing-style MoM components feature a sub-hemispherical bearing in which the coverage arc varies with bearing size. Devices with smaller bearing sizes have a lower coverage arc and are thus more prone to edge loading if implanted too steeply [26]. While most large-bearing MoM THR were withdrawn from the market for excessive ARMD, many resurfacing versions reported desirable outcomes [16–19]. Though large-bearing MoM THR devices provided excellent stability, the large head/neck ratio places too much stress on the mixed-metal taper connection, resulting in trunnion corrosion failure [27, 28]. If a trunnion could be designed to adequately handle the torque forces produced by the large head, perhaps a THR that combines a low wear bearing, excellent stability, and minimal trunnion corrosion risk could be achieved. We suggest this combination exists in the Magnum bearing with its large titanium (Ti) alloy neck adapter.

To this end, we present mid- to long-term clinical outcomes on this series of 211 cases with the uncemented Magnum large-bearing MoM THR. We describe our experience with the device and discuss some of the features that distinguish it from other MoM THR designs.

## Materials and methods

Between December 2004 and January 2016, the surgeon (TPG) implanted 211 Magnum THRs in 181 patients. The final Magnum THR case was performed in January 2016, after which the implant was withdrawn from the market due to higher failure rates of MoM THR. We retrospectively analyzed prospectively collected data from this consecutive series of Magnum cases. Patient demographics are listed in Table 1.

**Table 1:** Demographics

Variable	Result
Date range	12/2004–1/2016
# Of cases	211
# Deceased*	77 (36.5%)
*Demographics*	
% Female	119 (56.4%)
Mean follow-up (years)	10.1 ± 3.5
Age (years)	59.4 ± 11.6
BMI	31.4 ± 8.7
T-Score	− 0.6 ± 1.3
*Diagnoses*	
Osteoarthritis	139 (65.9%)
Dysplasia	12 (5.7%)
Rheumatoid arthritis	2 (0.9%)
Posttrauma	3 (1.4%)
Legg–Calve–Perthes disease	1 (0.5%)
Slipped capital femoral epiphysis	3 (1.4%)
Osteonecrosis	44 (20.9%)
Other	7 (3.3%)

The surgical procedure utilized a minimally-invasive posterior approach similar to that previously described for HRA [29]. However, after the capsulotomy was performed, the femoral neck was transected, and the acetabular component was placed. Before 2008, a 1-mm underream was used. Later, a wedge-fit technique was employed to reduce the rate of acetabular ingrowth failure [30]. Beginning in 2009, intraoperative radiographs were obtained to ensure placement of the acetabular component within relative acetabular inclination limit (RAIL) guidelines [31] to prevent adverse wear-related failure (AWRF).

The bulk material of the Magnum cup and head was made of an “as-cast” high carbon (> 0.2%) non-heat-treated cobalt (Co)-chromium (Cr) alloy. The bone ingrowth surface of the cup was plasma sprayed with a porous Ti alloy coating. Four pairs of small peripheral fins were placed on the exterior of the cup for increased initial stability. The cup had a thickness of 6 mm at the dome and a thickness of 3 mm at the rim to maximize bearing size while still preventing deformation during impaction. The coverage arc varied from 156° for the smallest bearing size (40 mm) to 164° for the largest bearing size (60 mm). This was the design feature that made the smaller components more prone to edge loading and subsequent metallosis [31], which we largely avoided by following the RAIL guidelines. The interior was highly polished to achieve a spherical tolerance of 200 µm. Magnum heads were made of the same Co-Cr and were also honed and buffed to the same spherical tolerance. The radial clearance of the bearing was 75–150 µm.

**Fig. 1:**
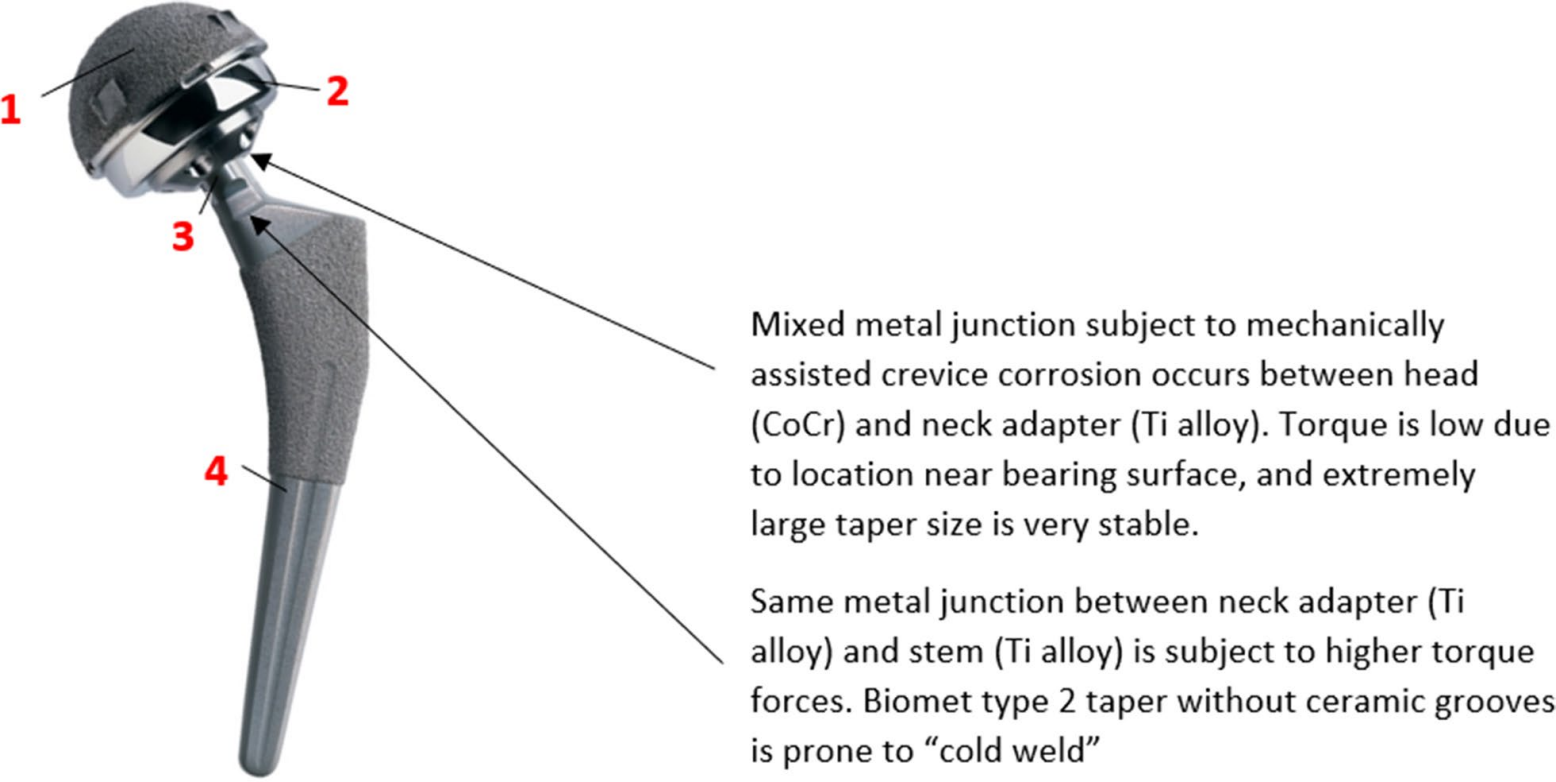
Biomet Magnum MoM THR: Design Rationale for Trunnion-Corrosion Resistance. The Biomet Magnum™ arthroplasty system is comprised of the: (1) Magnum Acetabular Component (Cast CoCr with Ti alloy plasma-spray coating), (2) Magnum Head (Cast CoCr), (3) Magnum Neck Adapter (Ti alloy), and (4) Taperloc Stem (Ti alloy)

Most THR feature modular heads of different lengths that are fixed to the stem trunnion by a morse cone taper junction. Large-bearing MoM designs typically comprise a Co-Cr head and Ti stem. These Ti stem trunnions have become smaller over time to increase head/neck ratio and improve stability in typical small-head systems [32, 33]. In addition, most manufacturers have modified the surfaces with grooves to comply with ceramic head manufacturers requirements. In most large MoM designs, a large Co head connects to a small Ti trunnion. In some cases, a small Ti or Co-Cr sleeve is interposed to adjust length. Therefore, very large torque forces are seen at the mixed-metal connection, occasionally causing trunnion corrosion by a mechanism known as mechanically assisted crevice corrosion. Figure 1 illustrates how this Magnum THR design differs; the large Ti neck adapter is interposed between the Ti trunnion and Co-Cr head. The mixed-metal junction was a larger taper connection situated near the bearing surface where it would be subject to minimal torque. The smaller taper junction between the adapter and the stem was a Ti-to-Ti connection. Also, the Biomet type 2 taper was larger than most on the market and was not modified for ceramic heads. We believe these design features made the Biomet design less prone to trunnion corrosion. Lavigne et al. [34] previously observed lower ion release with this design and hypothesized the same cause.

Full-weight bearing as tolerated was allowed immediately after surgery. Crutches were recommended for 1 to 2 weeks and a cane for 1 to 2 weeks thereafter. Physical therapy was limited to 1 to 2 days of in-hospital instruction. Extreme bending, leg crossing, heavy lifting, and high-impact activities such as jumping and jogging were discouraged until 6 months post-surgery. Deep-vein thrombosis precautions included sequential compression devices started intraoperatively and discontinued at the time of hospital discharge (mean 1.8 days postoperatively) as well as a variety of chemoprophylaxis agents.

We requested patients return for follow-up at 6 weeks, 1 year, 2 years, and then every other following year. Patients could come into the office for follow-up or complete them remotely. We requested a completed questionnaire and X-rays at each follow-up. These questionnaires facilitated data-collection for calculating Harris Hip score (HHS) [35], University of California, Los Angeles (UCLA) activity score [36], and visual analog scale (VAS) pain score for normal and worst days [37]. The HHS was used to determine clinical outcome; UCLA activity scores measured activity level after surgery on a scale from 1 to 10, for which 10 represented the highest level of activity; and VAS pain scores rated the level of pain from 0 to 10, with zero representing no pain and 10 representing maximum. Anterior–posterior pelvis and lateral radiographs were analyzed for component position, shifting, and radiolucencies by the surgeon (TPG). A physical examination was recorded on patients for the 6-week and 1-year follow-up as well as each in-office follow-up thereafter postoperatively to assess range-of-motion (ROM) and strength. At the 2-year follow-up, we requested that patients obtain a metal ion test (MIT). All data were prospectively collected in our clinical database. We were able to maintain a 90% rate of up-to-date follow-up. The average length of follow-up 10.1 ± 3.5 years (max 18 years). Metal ion tests were available in 68.2% of cases.

All statistical analyses were performed using the XLSTAT (Addinsoft, New York, NY) at a 95% confidence interval. We identified significant differences between group means using paired, 2-tailed Student’s t-test and between rates using two-sample proportion Z-tests.

## Results

**Fig. 2:**
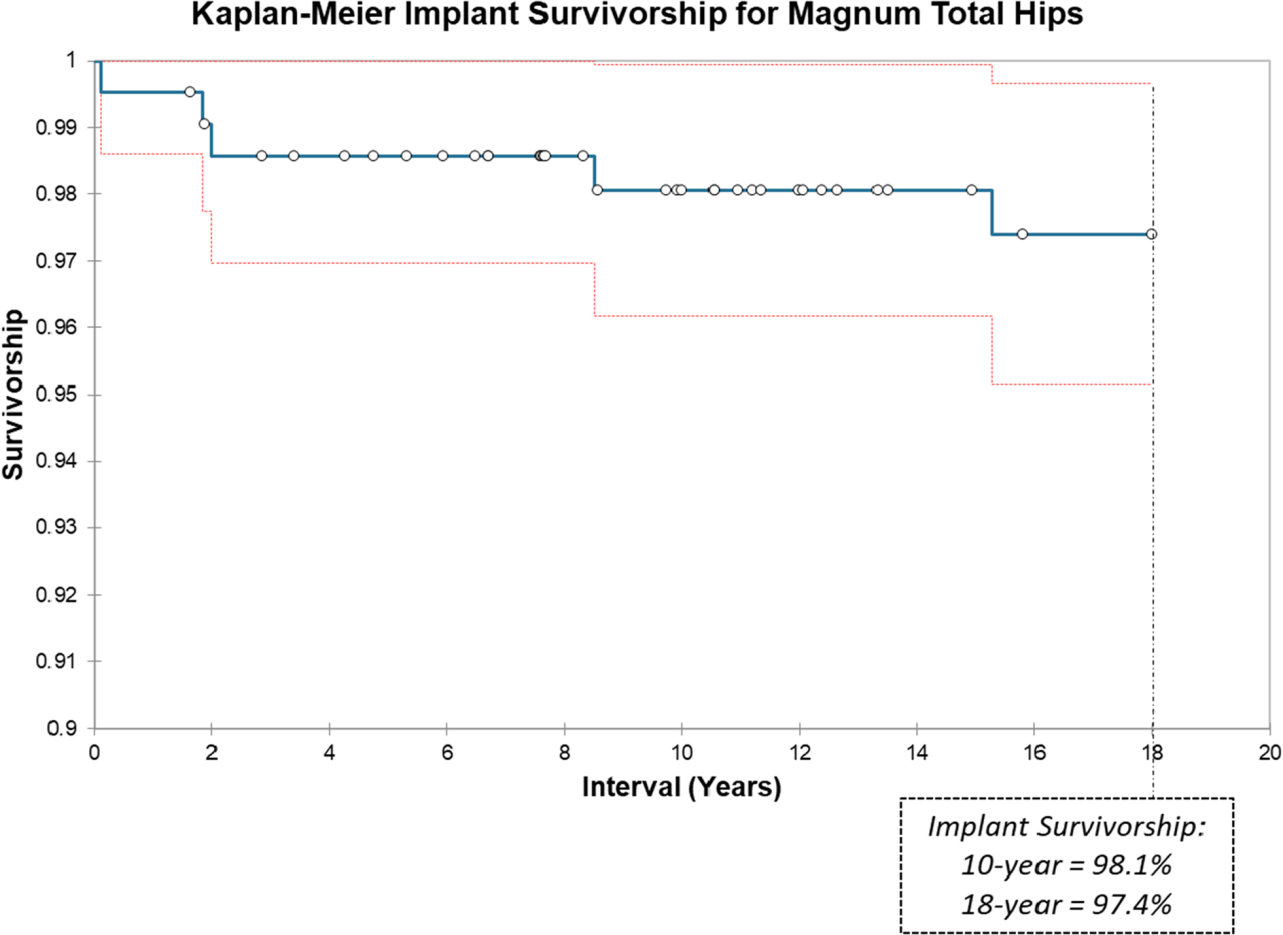
Kaplan–Meier Implant Survivorship for 211 Magnum Total Hips. Kaplan–Meier implant survivorship shown for this Magnum THR single-surgeon cohort at 95% confidence interval using revision of any component as endpoint

Kaplan–Meier (KM) implant survivorship was 98.1% at 10 years and 97.4% at 18 years postoperatively, using any revision as the endpoint (Fig. 2). There were eight total failures (3.8% of cases), one case of AWRF (0.5%; 1.8 years postoperatively), two failures of acetabular bone ingrowth (0.9%; 1.9 years and 2 months postoperatively), three trunnion corrosions (1.4%; 9.5, 10.6, and 15.3 years postoperatively), one late infection (0.5% at 9 years postop), and one inappropriate revision of implants for a trochanteric nonunion (0.5%) (Table 4).

The other trochanteric fracture was successfully repaired (0.5%). There was one successful reoperation for early infection at 6 weeks. There was one nonrecurrent dislocation, but no failures due to instability. Complications not requiring revision occurred in 12 cases (5.7%) (Table 5). Surgical results are listed in Table 2 and clinical outcomes in Table 3. There were no differences in the clinical scores (*p* = 0.1) or rate of revision between men and women (*p* = 0.7).

**Table 2:** Surgical results

Variable	Result
Length of incision (in)	3.5 ± 0.7
Operation time (min)	97.4 ± 21.3
Estimated blood loss (mL)	220.0 ± 156.6
Hospital stay (days)	1.8 ± 1.1
# Transfusion received	0 (0.0%)
ASA score	2.3 ± 0.6

**Table 3:** Clinical outcomes

Variable	Average
*Preoperative*	
HHS Score	46.5 ± 14.7
*Postoperative*	
HHS Score	94.5 ± 8.0
HHS pain Score	41.3 ± 6.1
UCLA Score	5.6 ± 1.8
VAS^2^ pain: regular	0.5 ± 1.1
VAS pain: worse	1.9 ± 2.5
Combined ROM	262.2 ± 28.2

There were four fractures (1.9%) within our Magnum THR cohort; this was higher than the 0.6% we reported for hip resurfacing. The first was a calcar femoral fracture 2 weeks postoperative. The stem subsided 1.5 cm before it stabilized and gained solid ingrowth with resultant leg-length inequality. This patient had their opposite hip replaced, reducing some of the length inequality. Despite their right Magnum hip still being shorter, the HHS was 100 at 13 years postoperatively. There were two trochanteric fractures repaired. One healed, and one failed before having well-fixed implants revised elsewhere. There was one trochanteric tip avulsion which was observed with a good outcome (Tables 4 and 5).

**Table 4:** Failures

Type	Result (#, %)
# Cases	211
*1) Acetabular failures*	
Adverse wear	1 (0.5%)
Acetabular component loosening	0 (0.0%)
Failure of acetabular ingrowth	2 (0.9%)
Fracture	0 (0.0%)
Acetabular component shift	0 (0.0%)
*2) Femoral failures*	
Femoral/trochanter fracture	1 (0.5%)
Femoral component loosening	0 (0.0%)
*3) Other failures*	
Trunnion corrosion	3 (1.4%)
Late infection	1 (0.5%)
Recurrent instability	0 (0.0%)
Unexplained pain	0 (0.0%)
Total failures	8 (3.8%)

**Table 5:** Complications/reoperations

Type	Result (#, %)
# Cases	211
*Complications*	
Acetabular component shift	2 (0.9%)
Dislocation	1 (0.5%)
Fracture	2 (0.9%)
Pulmonary embolus	1 (0.5%)
Sciatic nerve palsy	1 (0.5%)
Spinal headache	1 (0.5%)
*Reoperations*	
Deep infection (6 weeks)	1 (0.5%)
Fracture	2 (0.9%)
Unexplained pain	0 (0.0%)
Total	12 (5.7%)

There were 4 failures due to ARMD. One was a case of AWRF due to acetabular malposition discovered at 2 years. In this case, symptoms were mild, but the cup was excessively inclined (65°), ion levels were unacceptable (Co was 109.8 µg/L and Cr was 77.2 µg/L). Surgical findings included white fluid, gray stained tissues, and no muscle damage or osteolysis. The thickened gray lining was carefully removed. The cup and head were revised to another Magnum bearing, placed within the RAIL guidelines. The trunnion was normal. Within 6 weeks post-revision, ion levels normalized and the patient continues to do well 10 years later.

The three remaining ARMD cases displayed trunnion corrosion with distinctly different presentations. These were discovered ranging from 9 to 15 years postoperative, each patient presenting with significant pain. Ion levels were optimal for all 3 cases of trunnion corrosion prior to revision surgery. Cup positions were all within the RAIL guideline indicating low chance of edge loading. There were also large fluid collections on MRI. Surgical findings included large collections of brown fluid and tissue suggestive of chronic bleeding. The large Co-Ti trunnion was easily detached and looked normal. The smaller Ti–Ti trunnion was cold-welded and had significant black debris when separated. There was no gray metal staining of the tissues as seen in the bearing wear failure above and those that we have observed in hip resurfacing AWRF cases. Two of these patients were on chronic anticoagulation.

We performed an analysis of how radiographic cup position influenced ion levels and AWRF [31]. By 2009, we developed an acetabular component safe zone (known as RAIL guidelines). After this year, all cups met RAIL criteria and no AWRF occurred; prior to this year there was one AWRF due to acetabular component malposition. We compared ion levels between those that did and did not meet RAIL. Of the 211 Magnum patients, 144 (68.2%) complied with the request to obtain a MIT at 2 years postoperative or beyond. Tables 6 and 7 summarize MIT results. All values were converted to whole blood levels. Of the 211 cases, 189 had measurable x-rays. We found that 25 of these cases were over RAIL (13.2%) by an average of 9.9°. One of these cases failed due to AWRF (4%). Of the 25 cases over RAIL, 21 had MIT results. The AWRF case had high levels of whole blood metal ions, but the mean of the remainder was not significantly different from mean Co and Cr levels for cases under RAIL. This confirms our previous reports using the Magnum bearing in hip resurfacing; only approximately 5% of cases which fail to meet the RAIL safe zone develop metallosis, no cases that meet the RAIL criteria fail due to metallosis.

**Table 6:** MIT results

	Unilateral (*N* = 98)	Bilateral (*N* = 46)
Co* (µg/L)	1.7 ± 1.7	1.9 ± 1.3
Cr* (µg/L)	1.2 ± 1.4	1.0 ± 0.8
#, % Patients tested	144/211 (68.2%)	
#, % Levels converted	30/98 (69.4%)	22/46 (47.8%)
Normal (#, %)	74 (75.5%)	37 (80.4%)
Optimal (#, %)	94 (95.9%)	46 (100%)
Acceptable (#, %)	4 (4.1%)	0 (0.0%)
Problematic (#, %)	0 (0.0%)	0 (0.0%)
Toxic (#, %)	0 (0.0%)	0 (0.0%)

Failures excluded

**Table 7:** MIT Reference

	Normal^1^	Optimal^2^	Acceptable^3^	Problematic^3^	Potentially Toxic^2^
Unilateral Co Cr	< 1.5 µg/L < 1.5 µg/L	< 4.0 µg/L < 4.6 µg/L	4–10 µg/L 4.6–10 µg/L	10–20 µg/L 10–20 µg/L	> 20 µg/L > 20 µg/L
Bilateral Co Cr	< 1.5 µg/L < 1.5 µg/L	< 5.0 µg/L < 7.4 µg/L	5–10 µg/L 7.4–10 µg/L	10–20 µg/L 10–20 µg/L	> 20 µg/L > 20 µg/L

## Discussion

This study of 211 Magnum THR cases shows desirable clinical outcomes at mid- to long-term follow-up. KM implant survivorship was 98.1% at ten years and 97.4% at 18 years postoperative. With respect to implant survivorship, this surpasses the benchmark British NICE (National Institute for Clinical Excellence) criteria from 2014, which are set at 95% 10-year survivorship [38]. It also compares favorably to all three major joint registries [2–4] where 10-year THR implant survivorship averages at 94–95% with a mean patient age of approximately 70 years (compared to our cohort’s mean age of 59 years). In younger age groups, registry-reported 10-year implant survivorship values decrease. Further, survivorship in registries improves when excluding diagnoses other than osteoarthritis (OA). We include all diagnoses; 34% of our patients underwent surgery due to a diagnosis other than OA. The Australian registry [2] reports a 92% 15-year survivorship for a subset of only OA patients at mean age of 60 years.

Trunnion corrosion is still poorly understood. It is characterized by mild elevations in Co, large inflammatory brown fluid collections with extensive brown inflammatory tissue with the appearance of a chronic hematoma, occasional severe muscle damage, and no gray (Co) or black (Ti) metal staining. It occurs with all THR types but seems more common with a Co-to-Ti trunnion. The rate of trunnion corrosion failures for non-MoM THR bearing types is not truly known. One report by Hussey [39] indicated a 3.2% rate of trunnion corrosion failure for Zimmer brand THR using a Ti stem and a small Co-Cr head; no time to failure was presented. Most reports on THR include no data on trunnion corrosion. There is also no data available in the major registries [12, 32, 33]. In our cohort, the ion levels in trunnion failures (mean Co 4.1 ± 2.7, range 1.5–6.8) were typically well below what we experience with AWRF in hip resurfacing (mean Co 66.6 ± 50.1, range 13.0–152.6) [40]. We have seen no cases of AWRF with whole blood Co ion levels less than 13 µg/L. In Hussey’s report, levels of Co in trunnion corrosion were always below 10 µg/L and frequently below 4 µg/L. Therefore, despite mildly elevated Co levels in trunnion corrosion cases, we suspect some other yet unidentified moiety is responsible for the tissue damage seen in trunnion corrosion cases.

Most technical lessons pertaining to the Magnum acetabular component we learned from our larger experience of using this implant in MoM HRA (> 5000 cases) [41, 42]. We initially made the common mistake of implanting the resurfacing-style Magnum cup with the same method employed for metal-on-polyethylene THR cups. Since resurfacing-style MoM THR systems seem to require some variation from traditional THR surgical methods, incorrect technique could negatively bias the results of these systems and contribute to the worse outcomes seen with these implants. In the literature, the Biomet Magnum seems to have the best outcomes of the large-bearing MoM THR devices [2, 43], but the implant survivorship still lags behind most other THR systems [2].

A report from the Finnish registry indicated a 97% 4-year survivorship in 5464 uncemented Magnum THR [43]. Most other available reports on the MoM Magnum THR have been small series with short-term outcomes; these are summarized by Lombardi [44]. The two largest series to our knowledge were by Meding [45] and Lombardi [44]. Meding reported on 681 Magnum THRs with 2 to 5-year follow-up with 3 failures of cup ingrowth (0.4%), no cases of ARMD, and no dislocations. Lombardi reported a larger series of 1440 MoM THR with a 12-year 87% KM survivorship overall, although only 804 of these cases were with the Magnum. In their Magnum cohort of 804 cases, there were 19 (1%) ARMD failures and no cases of dislocation. Approximately 40% of the ARMD failures in the overall series had evidence for trunnion corrosion, which was hypothesized to be an additional source of metal debris. Steep cup position (> 55°) and female sex were risk factors. A longer-term series of Magnum THAs published more recently by Lombardi et al. [44] shows similar outcomes, reporting 88.6% implant survivorship at 16 years for any cause revision.

Numerous studies on the Metasul 28-mm show excellent implant survivorship, ranging from 94 to 98% at 7–15 years with few ARMD failures. The Metasul 28-mm MoM articulation has a full hemisphere cup (180°) and a small-head-neck ratio at the mixed-metal trunnion connection [46, 47]. Therefore, this implant is not prone to edge loading with subsequent AWRF or trunnion corrosion, but the issue of instability remains.

Modular Ti-on-Ti trunnions may exhibit cold welding, leading to difficulty of removing the neck adapter from the stem [48, 49]. All three cases (1.4%) of trunnion corrosion that we revised did present cold-welding at the time of revision surgery, though this degree varied. Specialized instruments should be available in the operating room during revision in case cold-welding is encountered. Biomet can supply these. Occasionally the trunnion cannot be separated, and a decision must be made to leave the head or remove the well-fixed stem.

There are several design limitations to this study worth discussing. First, the variation in demographics from average arthroplasty cohorts makes comparison difficult; when compared with registry populations, our study group had fewer cases of OA and had a younger mean age. Comparison is further made difficult as the MoM Magnum total hip device is no longer on the market. Thus, future prospective studies with this implant would not be possible. However, we suggest the taper design of this system may make these results of particular interest in the future investigations of trunnion corrosion, which is still a poorly understood mechanism of failure in THR [48, 49]. Next, two of the three trunnion corrosion failures we diagnosed were on chronic anticoagulation. The large fluid collections had the appearance of chronic hematomas intraoperatively. We are currently uncertain what interplay, if any, exists between these trunnion corrosion collection and anticoagulation therapy.

## Conclusions

We present excellent 8- to 18-year implant survivorship for 211 cases of Biomet Magnum MoM (98.1% at 10 years and 97.4% at 18 years) exceeding the NICE criteria and major registry benchmarks. AWRF was present in one case with acetabular component malposition. By using the RAIL cup position guidelines, wear failure has been avoided in all cases after 2009. Trunnion corrosion caused failures in 3 (1.4%) cases at 9–15 years postoperative. This rate is comparable to other THR systems with similar bearing design. This study suggests MoM THR of comparable design could be a safe and viable hip arthroplasty option when performed by an experienced surgeon. AWRF is rare, and revision has similar outcomes as any other cause of failure. This paper demonstrates desirable outcomes in MoM THR with this unique trunnion connection, but we propose that application of these design principles in large head metal-on-polyethylene THR could be a valuable method in addressing problems of dislocation and trunnion corrosion that still afflict THR today.

## Data Availability

Summary data are available in the attached data tables. Raw data free of patient-identifying information is available upon request, or within the Synapse data repository (SynID: syn51522715). References to previous datasets are cited in relevant places within the text.

## Abbreviations

THR: Total hip replacement
MoM: Metal-on-metal
HRA: Hip resurfacing arthroplasty
ARMD: Adverse reaction to metal debris
Ti: Titanium
AWRF: Adverse wear-related failure
RAIL: Relative acetabular inclination limit
Co: Cobalt
Cr: Chromium
ROM: Range of motion
MIT: Metal ion test
KM: Kaplan–Meier
